# Sensitivity and Specificity of Interictal EEG-fMRI for Detecting the Ictal Onset Zone at Different Statistical Thresholds

**DOI:** 10.3389/fneur.2014.00131

**Published:** 2014-07-17

**Authors:** Simon Tousseyn, Patrick Dupont, Karolien Goffin, Stefan Sunaert, Wim Van Paesschen

**Affiliations:** ^1^Laboratory for Epilepsy Research, UZ Leuven and KU Leuven, Leuven, Belgium; ^2^Medical Imaging Research Center, UZ Leuven and KU Leuven, Leuven, Belgium; ^3^Laboratory for Cognitive Neurology, UZ Leuven and KU Leuven, Leuven, Belgium; ^4^Department of Nuclear Medicine, UZ Leuven and KU Leuven, Leuven, Belgium; ^5^Radiology Department, UZ Leuven and KU Leuven, Leuven, Belgium

**Keywords:** EEG-fMRI, refractory focal epilepsy, presurgical evaluation, sensitivity and specificity, interictal

## Abstract

There is currently a lack of knowledge about electroencephalography (EEG)-functional magnetic resonance imaging (fMRI) specificity. Our aim was to define sensitivity and specificity of blood oxygen level dependent (BOLD) responses to interictal epileptic spikes during EEG-fMRI for detecting the ictal onset zone (IOZ). We studied 21 refractory focal epilepsy patients who had a well-defined IOZ after a full presurgical evaluation and interictal spikes during EEG-fMRI. Areas of spike-related BOLD changes overlapping the IOZ in patients were considered as true positives; if no overlap was found, they were treated as false-negatives. Matched healthy case-controls had undergone similar EEG-fMRI in order to determine true-negative and false-positive fractions. The spike-related regressor of the patient was used in the design matrix of the healthy case-control. Suprathreshold BOLD changes in the brain of controls were considered as false positives, absence of these changes as true negatives. Sensitivity and specificity were calculated for different statistical thresholds at the voxel level combined with different cluster size thresholds and represented in receiver operating characteristic (ROC)-curves. Additionally, we calculated the ROC-curves based on the cluster containing the maximal significant activation. We achieved a combination of 100% specificity and 62% sensitivity, using a *Z*-threshold in the interval 3.4–3.5 and cluster size threshold of 350 voxels. We could obtain higher sensitivity at the expense of specificity. Similar performance was found when using the cluster containing the maximal significant activation. Our data provide a guideline for different EEG-fMRI settings with their respective sensitivity and specificity for detecting the IOZ. The unique cluster containing the maximal significant BOLD activation was a sensitive and specific marker of the IOZ.

## Introduction

The goal of the presurgical evaluation in refractory focal epilepsy is to define the epileptogenic zone, the area indispensable for the generation of epileptic seizures (1).

In the last decade, the value of simultaneous electroencephalography-functional magnetic resonance imaging (EEG-fMRI) as a localizing tool of the epileptogenic zone has been explored. In this technique, changes in blood oxygen level dependent (BOLD) contrast, related in a statistical way to interictal epileptic discharges or seizures, are displayed as spatial maps. Sensitivity of EEG-fMRI for localizing the epileptogenic zone has received a lot of attention, but specificity has largely been neglected (2), hampering the clinical implementation of EEG-fMRI in the presurgical evaluation of refractory focal epilepsy.

In order to address this issue, correlational studies of EEG-fMRI with a “gold standard” are crucial (3). Since the epileptogenic zone is a theoretical concept, the ictal onset zone (IOZ), the area from which seizures are generated, is a valuable alternative. The IOZ can be determined by ictal scalp/invasive EEG-registrations and/or ictal single photon emission computed tomography (SPECT) in concordance with other presurgical investigations (1).

Several validation studies assessed the sensitivity of spike-related EEG-fMRI using the results of ictal invasive EEG-registrations as indication of the IOZ (4–17). A major disadvantage of intracranial EEG is the limited spatial coverage and the necessity of an *a priori* hypothesis of the IOZ with possible non-localizing or misleading results (18, 19). The IOZ is then determined by arbitrary margins around contact points, active during seizure onset, but the technique is blind to uncovered areas, a particular problem when comparing with BOLD activity, as experienced by several authors (5, 8, 17, 20–23).

Ictal perfusion SPECT has the advantage of demonstrating dynamic seizure-related changes in cerebral perfusion on a whole brain scale, which offers ideal comparison with fMRI studies. Several interictal EEG-fMRI studies used the results of ictal SPECT in their validation (5, 6, 9, 11, 12, 24–26). Due to its low temporal resolution, both the IOZ and seizure propagation pathways can be found (27–30). Therefore, combinations of imaging modalities [structural MRI, interictal and ictal SPECT and subtraction ictal SPECT co-registered to MRI (SISCOM), and interictal ^18^F-fluorodeoxyglucose positron emission tomography (^18^F-FDG PET)], which integrate the additional information of each independent modality, may provide superior information compared to the information provided by ictal SPECT alone (29).

More recently, spike-related EEG-fMRI results have been compared with postsurgical resection zone and epilepsy outcome (5, 9–12, 14, 16, 17, 21, 24, 26, 31, 32). After successful epilepsy surgery, sufficient brain tissue has been resected. However, the resection zone depends on the surgical approach (conservative versus extended resection) (33) and the accessibility and can overestimate the actual IOZ.

A much larger problem than the exact definition of the IOZ to define sensitivity of EEG-fMRI is the lack of knowledge about specificity of EEG-fMRI (2). The extent and pattern of the BOLD changes are dependent on the statistical threshold levels that are used. Less stringent threshold levels will not only induce more widespread and multifocal patterns but also more false-positive results.

At conventional statistical threshold levels [family-wise error (FWE) corrected *p* < 0.05 and uncorrected *p* < 0.001] both focal and multifocal, widespread BOLD responses have been described. Widespread BOLD signal clusters have been interpreted as representing widespread epileptic abnormalities with poor surgical prognosis if not completely included in the resection (10, 16, 21, 31). However, in the absence of knowledge about specificity of EEG-fMRI, the clinical significance of these different patterns remains uncertain. As mentioned by Chaudhary and colleagues in 2012, EEG-fMRI studies demonstrate “often complex BOLD patterns, raising the issue of specificity of the findings and the unknown clinical relevance of individual BOLD clusters” (34). In a paper of van Houdt and colleagues, this was rephrased as “there are currently no standards for the statistical thresholds in EEG-fMRI analysis” (17).

In this study, we propose an innovative approach to quantitatively define the effect of different statistical thresholds on sensitivity and specificity of spike-related BOLD changes for detecting the IOZ. We determined true-positive and false-negative BOLD fluctuations in patients, and false-positive and true-negative BOLD fluctuations in age- and gender-matched healthy case-controls.

## Materials and Methods

### Inclusion criteria

This study was approved by an independent ethical standards committee on human experimentation of the University Hospitals Leuven and written informed consent was obtained from all participants.

Inclusion criteria were (i) consecutive adults who underwent a full presurgical evaluation for refractory focal epilepsy between August 2010 and November 2013, including seizure history, neurological and physical examination, neuropsychological assessment, interictal and ictal scalp EEG-recordings, video-analysis of seizures, high-resolution MRI of the brain, and in most patients SISCOM and interictal ^18^F-FDG PET. In selected cases, intracranial EEG-recordings were performed; (ii) concordant data pointing to one epileptic focus using all available presurgical investigations, including a SISCOM or else successful outcome after epilepsy surgery [international league against epilepsy (ILAE) outcome classification 1–3 (1, completely seizure-free; 2, only auras; 3, one to three seizure days per year ± auras; 4, four seizure days per year to 50% reduction of baseline seizure days ± auras; 5, <50% reduction of baseline seizure days to 100% increase of baseline seizure days ± auras; 6, more than 100% increase of baseline seizure days ± auras)] (35); (iii) recording of interictal spikes during EEG-fMRI.

### Operational definition of the ictal onset zone

The IOZ was defined as follows:

(i) In patients with successful outcome after epilepsy surgery, we considered the manually outlined resected brain area as the IOZ.
(ii) In patients, awaiting surgery, refusing surgery, or ineligible for surgery due to proximity of the epileptogenic zone to eloquent regions, we determined the IOZ as the hypothetical resection zone, based on multidisciplinary clinical consensus using all non-invasive and invasive data except EEG-fMRI results. As the patients were selected for concordant localizing data, we ensured not to rely on a single testing modality. The volume of this IOZ was further restricted to the region of ictal hyperperfusion on SISCOM within this hypothetical resection area. The hyperperfusion was thresholded with a *Z*-score = 1.5. This threshold was shown to be optimal for localizing the epileptogenic zone (36).

### EEG-fMRI acquisition and processing

Functional images were acquired using a whole brain single-shot T2* gradient-echo Echo Planar Imaging sequence in one of two 3 T MR scanners (Achieva TX with a 32-channel head coil and Intera Achieva with an eight-channel head coil, Philips Medical Systems, Best, The Netherlands); TE = 33 ms, TR = 2.2 or 2.5 s, voxel size 2.6 mm × 3 mm × 2.6 mm.

A 64- or 32-channel MR compatible EEG cap was used for simultaneous EEG-fMRI recordings with a BrainAmp amplifier (Brain Products, Munich, Germany; sampling rate 5 kHz). In patients admitted to the hospital, we used a 24-channel MR compatible electrode set (Yves EEG solutions, Newburyport, MA, USA) both in the telemetry unit and in the scanner with the BrainAmp amplifier. Patients were asked to rest with closed eyes.

The EEG was filtered offline (bandpass 1–50 Hz) and gradient artifacts were removed using the Bergen plug-in (Bergen fMRI Group, Bergen, Norway)^1^ (37) for EEGLAB.^2^ Pulse artifacts were subtracted with Brain Vision Analyzer software (Brain Products, Munich, Germany) (38).

The fMRI data were analyzed with statistical parametric mapping (SPM), version 8 (Wellcome Department of Imaging Neuroscience, University College London, UK)^3^; running on MATLAB (MathWorks, Natick, MA, USA). Images were realigned, slice-time corrected, normalized into the Montreal Neurological Institute (MNI) space using the T1 MRI template available in SPM (voxel size: 2 mm × 2 mm × 2 mm), and spatially smoothed using an isotropic Gaussian kernel of 6 mm full width at half-maximum (FWHM).

Interictal spikes were visually marked by a neurologist (Simon Tousseyn) and discussed with a second neurologist (Wim Van Paesschen). Statistical analysis was performed using the general linear model approach. The regressor of the interictal spike was created using the timings of the event convolved with the canonical hemodynamic response function. Included as confounding covariates were (i) the six rigid-body motion correction parameters, (ii) the fMRI signal averaged over the lateral ventricles, and (iii) the fMRI signal averaged over a region centered in the white matter (39). When a sudden head movement (>1 mm translation) appeared, we added a dummy regressor, which was set to one for the corresponding scan as well as for the next three scans. The remainder of the regressor was set to 0. In case this sudden movement was present in different consecutive blocks, a dummy regressor was created for each block (40, 41).

A statistical *Z*-score map was obtained for the interictal spike event-related regressor. In case, a patient had more than one spike-type, only EEG-fMRI results corresponding to the most frequent spike-type, determined during video-telemetry, were used for the analysis.

### Sensitivity and specificity for localizing the ictal onset zone

**Figure 1:**
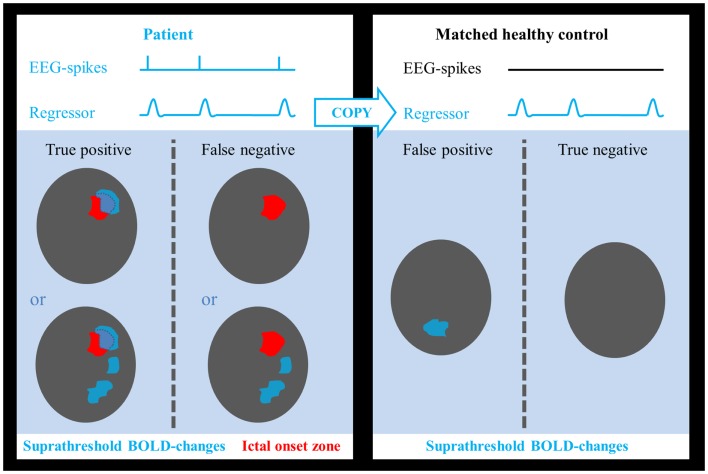
**Determination of test outcome**. EEG-spikes = spike-time course based on manually indicated interictal spikes. Regressor = spike-time course of patient convolved with canonical hemodynamic response function. Areas of suprathreshold BOLD changes overlapping the ictal onset zone in patients were considered as true positives and if no overlap was found, they were treated as false negatives. Suprathreshold BOLD changes in any part of the brain in healthy controls were considered as false positives, absence of BOLD responses as true negatives.

Sensitivity and specificity were calculated as follows: true-positive cases were defined as those patients in whom we found a suprathreshold cluster of a suprathreshold size overlapping the IOZ. Patients, in whom this was not the case, were considered false-negative cases. Epilepsy can be regarded as a network disorder (42, 43). This network concept implies interregional interactions between the IOZ and other sites. Based on this theory, we believe it is not appropriate to classify activations outside the IOZ, related to spikes in patients, as false positives. To determine false-positive and true-negative cases, we introduced age- and gender-matched healthy controls assigned to each patient in order to obtain a statistical map using the spike event-related regressor of that patient (corresponding to nonsense events for the control subject). False-positive cases were those controls who showed a suprathreshold cluster of a suprathreshold size somewhere in the brain while true-negative cases were those controls for whom this was not the case (see Figure 1). In a way, we treated the controls as a surrogate for the patient group, assuming that the results would have been the same if we had been able to look at those parts of the brain, which were not linked to the epileptic network. Each control underwent EEG-fMRI using the same session length as the corresponding patient. The spatial normalization step ensured that the number of voxels, which were analyzed, as well as the cluster size was similar between all patients and controls.

**Figure 2:**
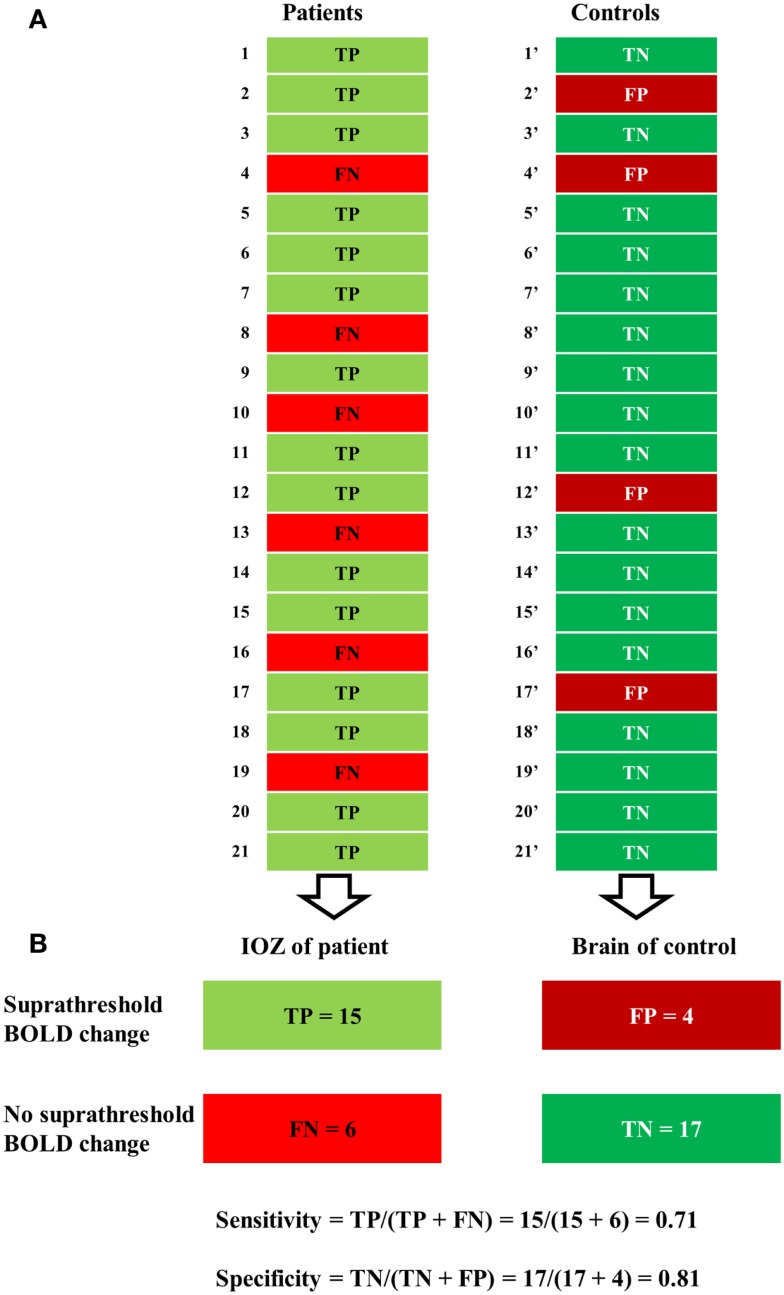
**Illustration of a contingency table**. **(A)** For a certain statistical threshold, true-positive (TP) or false-negative (FN) cases are determined in patients, false-positive (FP) or true-negative (TN) cases in control subjects. **(B)** Sensitivity (number of true positives over the number of true positives and false negatives) was defined solely in the patient group and specificity (number of true negatives over the number of true negatives and false positives) solely in the control group.

At a certain statistical threshold, sensitivity was defined as the proportion of true-positive cases within the patient group and the “surrogate” specificity as the proportion of true-negative cases within the control group (see Figure 2). Sensitivity and specificity were calculated for different statistical thresholds at the voxel level (*Z*, 0–13; step-size, 0.1). This was combined with different thresholds for the minimal cluster size up to 600 voxels (step-size, 50 voxels, voxel size, 2 mm × 2 mm × 2 mm). Six hundred voxels correspond to a brain volume of 4800 mm^3^, comparable to the volumes of a hippocampus (44), or a focal cortical dysplasia (27). Based on these results, receiver operating characteristic (ROC)-curves were calculated. We performed the calculations for activations and deactivations, separately.

In an attempt to identify the most localizing cluster of BOLD changes, if widespread BOLD changes were present, several authors looked at the cluster containing the maximal significant activation or at the maximal significant activation voxel (45, 46). In an additional analysis, we calculated the ROC-curves based upon each of these selections.

## Results

### Study population

Twenty-one consecutive patients (age: 36 ± 14 years, age at epilepsy onset: 12 ± 10 years, 16 women) met the inclusion criteria. Clinical data are presented in Table 1. Patients had a median seizure frequency of eight seizure days per month (range 0.5–30) and had failed 7 ± 4 antiepileptic drugs at the time of evaluation. Thirteen patients had temporal lobe epilepsy (TLE): six mesial TLE, seven lateral TLE, while seizure onset was extratemporal (ETLE) in the other eight patients. Structural imaging was normal in 3/21 patients. All ictal SPECT injections used for analysis in this study were performed during the ictal phase [median seizure duration, 84 s (range: 5–423); median time of initiation of ictal SPECT tracer injection, 17 s (range: 1–43)]. Intracranial EEG-recordings, available in five patients, were concordant with non-invasive investigations.

**Table 1:** **Clinical data of patients**.

Patient	Gender/age/age at onset	Ictal onset zone	Etiology	Symptomatogenic zone	Structural MRI	SISCOM localization	Interictal ^18^F-FDG PET hypometabolism	Scalp interictal spike localization^§^	Scalpictal EEG localization	icEEG	Epilepsy surgery	ILAE outcome	Follow-up time after surgery (m)	Pathology	Spike-rate (spikes/h) during fMRI
1	F/48/3	R insula	Unknown	Insula	Normal	R insula	Not contributive	F4	R frontal	Concordant	Planned				384
2	F/38/12	R temporal	CNS infection	R temporal	Ischemic R temporoparieto-occipital lesion	R temporal	Not done	T4	R	Not done	Refusal				1171
3	F/23/9	L anterior temporal	DNET	L temporal	L temporal DNET	L temporal	L temporal	F7 T1	L temporal	Not done	Yes	2	27	DNET	187
4	F/56/8	R parietooccipito-temporal	Sturge-Weber	R temporal	Superficial angioma and hemiatrophy R posterior convexity	R parietooccipito-temporal	Not done	F8	R frontocentral	Not done	Planned				156
5	M/20/7	L temporoparietal	Unknown	Temporal	L parietal gliosis after surgery	L temporoparietal	Not done	T5	L posterotemporal	Concordant	Overlap eloquent cx				15
6	F/34/15	R frontal	FCD	R extratemporal	R frontal FCD	R frontal	R frontal	Cz C4	R centroparietal	Concordant	Yes	2	22	Gliosis, neuronal loss, microglia activation	853
7	F/55/38	L anterior temporal	HS	L temporal	L HS	L temporal	L temporal	F7 T1	L temporal	Not done	Yes	1	27	HS	156
8	F/61/12	L frontal	FCD	L frontal	L frontal FCD	L frontal	Not done	C3 Cz	L frontocentral	Not done	Overlap eloquent cx				915
9	M/29/27	R frontal	FCD	R frontal	R frontal FCD	R frontal	Not done	F4	R frontocentral	Concordant	Yes	1	5	FCD	64
10	M/30/15	R temporal	Unknown	Temporal	Normal	R temporal	Not done	F8 T4	R temporal	Not done	Planned				733
11	F/23/2	L temporal	FCD	Temporal	L temporal FCD	L temporal	L temporal	F7 T1	L temporal	Not done	Refusal				181
12	F/23/9	L parietooccipital	Perinatal infarction	Extratemporal	L medial cerebral artery infarction	L parietal	L parietooccipital	O1	Midcentral	Not done	Yes	1	2	No significant abnormalities	398
13	F/33/6	L temporooccipital	FCD	L posterior quadrant	L posterior temporal FCD	L occipitotemporal	L occipital	T3 T5	L posterior	Not done	Overlap eloquent cx				1613
14	M/16/7	R frontal	FCD	R frontoparietal	R mesial frontal FCD	R frontal	Frontoparietal	Cz	R and midcentral	Concordant	Overlap eloquent cx				2018
15	F/40/1	L parietal	FCD	Extratemporal	L parietal FCD	L parietal	Not done	Pz	L and midcentral	Not done	Overlap eloquent cx				589
16	F/43/12	L anterior temporal	HS	L temporal	L HS	L temporal	L temporal	F7 T1	L temporal	Not done	Yes (radiosurgery)	1	10	Not available	73
17	F/30/14	R temporal	Ganglioglioma	R temporal	R temporal ganglioglioma	Not done	R temporal	F8	R temporal	Not done	Yes	1	8	Ganglioglioma	115
18	M/20/8	L anterior temporal	HS	Temporal	L HS	Not done	L temporal	F7 T1	L temporofrontal	Not done	Yes	1	3	HS	8
19	F/45/19	R temporal	Unknown	R temporal	Normal	R temporal	R temporal	F8 T2	R temporal	Not done	Refusal				168
20	F/56/32	R temporal	HS	R temporal	R HS	R temporal	R temporal	F8 T2	R temporal	Not done	Refusal				602
21	F/30/0	L temporal	HS	L temporal	L HS	L temporal	L temporal	F7 T1	L temporal	Not done	Planned				40

*SISCOM, subtraction ictal SPECT co-registered to MRI; ^§^international 10–20 EEG-system (additional T1 and T2 electrodes); icEEG, intracranial EEG-recordings; ILAE outcome (35) for available follow-up, F, female; M, male; R, right; L, left; CNS, central nervous system; DNET, dysembryoplastic neuroepithelial tumor; FCD, focal cortical dysplasia; HS, hippocampal sclerosis; cx, cortex*.

So far, eight patients underwent epilepsy surgery with a successful outcome [ILAE class 1 (completely seizure-free) in six cases, class 2 (only auras) in two cases] (35) (median follow-up time, 9 months; range, 2–27): temporal lobe resection (patients 3, 7, 17, and 18), frontal lesionectomy (patients 6 and 9), functional hemispherotomy (patient 12), and hippocampus/amygdala radiosurgery (patient 16).

Functional magnetic resonance imaging sessions lasted on average 49 ± 15 min. The median spike-rate during EEG-fMRI was 187 spikes/h (range: 8–2018). Twenty-one healthy case-controls (age, 36 ± 12 years; 16 women) underwent EEG-fMRI using the same session length.

### Sensitivity and specificity for localizing the ictal onset zone

**Figure 3:**
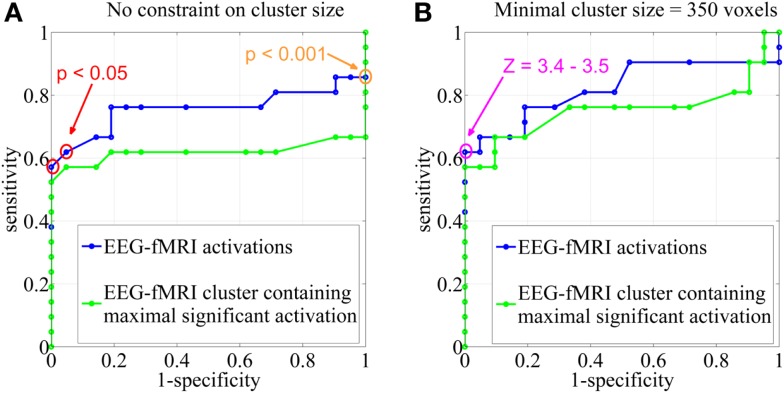
**Sensitivity and specificity**. Receiver operating characteristic (ROC)-curves using different statistical *Z*-score thresholds at the voxel level (range, 0–13; step-size, 0.1) and **(A)** no constraint on cluster size, **(B)** a minimal cluster extent of 350 voxels. The results were based on all EEG-fMRI activations (blue) and upon the cluster containing the maximal significant activation only (green). The orange circle corresponds to the statistical threshold of uncorrected *p* < 0.001 (*Z* = 3.1), red circles to a significance level of FWE corrected *p* < 0.05 (*Z* = 4.9–5.1). The pink circle represents *Z*-score thresholds of 3.4 and 3.5. Note that not every change in *Z*-score threshold is associated with a change in sensitivity and specificity.

**Figure 4:**
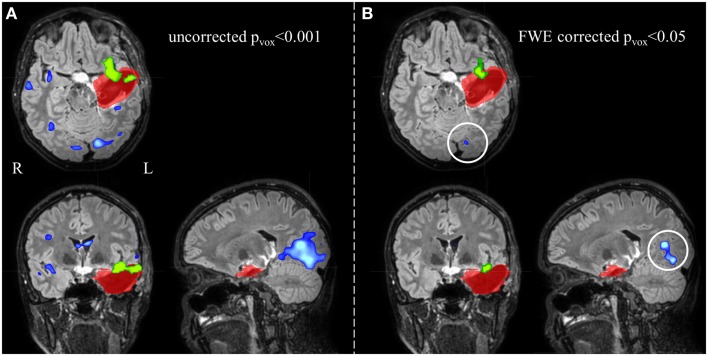
**Electroencephalography-functional magnetic resonance imaging activations are overlaid on representative slices of the postsurgical structural image of patient 3 (A) after thresholding at an uncorrected *p* < 0.001 (*Z* = 3.1, no constraint on cluster size) and (B) at a FWE corrected *p* < 0.05 (*Z* = 5.1, no constraint on cluster size)**. **(A)** The EEG-fMRI cluster containing the maximal significant activation (green colored) is overlapping the IOZ (red colored). Widespread additional suprathreshold activation clusters (blue colored) are present. At these settings, it is unclear whether these activations outside the IOZ represent false- or true-positive activations. **(B)** Using a more stringent statistical threshold (corresponding to 100% specificity), fewer activation clusters survive. The left occipital activation (white circle) is not a false-positive but a true-positive detection and is considered part of a spike-related network; R, right; L, left.

Electroencephalography-functional magnetic resonance imaging BOLD activations corresponding to the statistical threshold of uncorrected *p* < 0.001 (*Z* = 3.1, no constraint on cluster size) resulted in 86% sensitivity (suprathreshold activations in the IOZ in 18 of the 21 patients) and 0% specificity (all controls had a suprathreshold detection in the brain). In contrast, when a significance level of FWE corrected *p* < 0.05 was used (corresponding to a *Z* between 4.9 and 5.1 in our study, no constraint on cluster size), sensitivity dropped to 62–57% but specificity increased to 95–100% (Figures 3 and 4).

**Figure 5:**
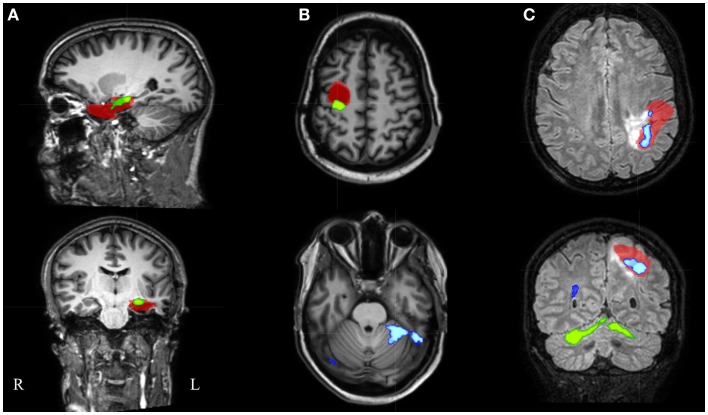
**Electroencephalography-functional magnetic resonance imaging results after thresholding at *Z* = 3.5 at the voxel level combined with a minimal cluster extent of 350 voxels are overlaid on representative slices of the structural MRI in three patients**. **(A)** Patient 16 had only 1 suprathreshold activation. This cluster containing the maximal significant activation (green colored) was localized within the IOZ (red colored). **(B)** In patient 6, the cluster containing the maximal significant activation (green colored) was overlapping the IOZ, while an additional activation cluster (blue colored) was situated within the contralateral cerebellum. **(C)** Patient 15 was the only patient with a BOLD activation cluster (blue colored) overlapping the IOZ, which did not contain the maximal significant activation (green colored); R, right; L, left.

We report the settings that give the highest sensitivity for maximal specificity. *Z*-score thresholds of 3.4 and 3.5 both resulted in 62% sensitivity and 100% specificity, using a minimal cluster size of 350 voxels (Figure 3). At these settings, 6 of the 13 patients (46%) with an overlap between a cluster of BOLD activation and the IOZ had at least one additional activation cluster in more remote areas, not overlapping the IOZ. Exclusion of these remote activations from resection did not preclude successful outcome in three of the eight operated patients (patients 3, 6, and 16). In patient 3, a dysembryoplastic neuroepithelial tumor (DNET) in the left temporal lobe was resected. Remote activations were mainly located in mesial occipital areas. Patient 6, with a focal cortical dysplasia in the right primary motor cortex, had a contralateral cerebellar activation (Figure 5). Successful radiosurgery involved the left hippocampus in patient 16 with left hippocampal sclerosis. Additional BOLD activations were present in the left temporal neocortex.

In an additional analysis, we looked at the cluster containing the maximal significant BOLD activation. This resulted in similar sensitivity and specificity when using the same minimal cluster extent of 350 voxels (Figure 3). A sensitivity of 57% and a specificity of 100% were obtained for a broad range of *Z*-score thresholds from 3.2 to 3.5 (green areas in Figure 5). Interestingly, this combination of sensitivity and specificity could also be reached using other settings for minimal cluster size (from 250 voxels up to 600 voxels) but with a smaller range of corresponding *Z*-score thresholds. By using the cluster containing the maximal significant activation instead of all EEG-fMRI activations, we discarded the additional, non-localizing clusters (blue areas in Figure 4) distant from the IOZ with sacrificing minimal sensitivity (4.8%) at 100% specificity. Only 1 of 21 patients (4.8%) (patient 15) showed a BOLD activation cluster overlapping the IOZ, which did not contain the maximal significant activation (Figure 5).

Finally, when using the maximal significant activation voxel or when considering BOLD deactivations, an overall low performance was found.

## Discussion

Electroencephalography-functional magnetic resonance imaging has evolved from a research tool and is on the brink of becoming a clinical method to delineate the ictal onset in the presurgical evaluation of patients with refractory focal epilepsy. Before taking decisions based on EEG-fMRI, validation studies are a prerequisite. We felt that it was important to investigate sensitivity and specificity of interictal EEG-fMRI for localizing the IOZ, in those patients in whom spikes were visually detected during EEG-fMRI. A reliable test combines a high sensitivity with a high specificity.

### Specificity

The setting of an appropriate statistical threshold in functional imaging is a critical point (6, 32, 46, 47). EEG-fMRI validation studies focused on sensitivity for detecting the IOZ, using “standard” statistical thresholds, but the specificity of these results is still largely unknown. fMRI responses exceeding the epileptogenic zone are often reported (17, 48, 49). A possible explanation may be the choice of a low statistical threshold resulting in false-positive responses (25) and understanding how to minimize these false positives would be of great interest (50). On the other hand, it is not excluded that hemodynamic changes outside the IOZ are related to an epileptic network. Hence, it would not be appropriate to consider hemodynamic changes, associated to spikes in patients and localized partially within and partially outside the IOZ, as false positives. To tackle this issue, we chose to determine false-positive and true-negative cases in age- and gender-matched healthy case-controls. The interictal spike event-related regressor of the patient was used in the design matrix of the healthy case-control (corresponding to nonsense events for this control subject). False-positive cases were those controls who showed a suprathreshold BOLD change in the brain while true-negative cases were those controls for whom this was not the case. As argued before, we treated controls as a surrogate for the patient group with the assumption that results would have been the same if we were able to look at those parts of the brain that were not linked to the epileptic network. The surrogate specificity was subsequently determined as the proportion of true-negative cases within the control group. A disadvantage of our approach is the unknown contribution of differences in noise level between patient and control datasets.

Alternatively, false-positive and true-negative rates could be established in the patient group after random annotation of spike onsets (2). Specificity would then equal the proportion of patients who lack a suprathreshold BOLD fluctuation in the brain, related to these nonsense events. The calculation of true-positive and false-negative rates could remain unchanged, based on real spike onsets. This approach holds two potential risks. In patients with high spike rates, coincidental correlation between regressors related to nonsense events and to real spikes becomes more likely, causing an overestimation of false-positive rates. This problem does not apply in healthy volunteers. A second risk is related to the poorly understood occurrence in time of epileptic spikes. It is not excluded that this occurrence follows a rhythmic pattern, which exhibits (whether or not coincidental) temporal similarities with activity fluctuations of normal physiological brain processes. In that case, random assignment of spike onsets would break this rhythmicity and potentially cause an underestimation of false-positive rates. When we copy the regressor-of-interest to healthy volunteers, this rhythmicity remains unmodified. Notwithstanding the concerns of the alternative method described above, permutation of original spike onsets led to similar sensitivity and specificity (see alternative approach, included as Supplementary Material).

To the best of our knowledge, only three papers formally addressed the topic of specificity of interictal EEG-fMRI in a quantitative way. First, Waites and colleagues used a non-parametric permutation approach in two patients with childhood absence epilepsy and one healthy control to investigate if interictal discharges lead to a BOLD response that is significantly different from chance (2). It was shown that “activations” (at a corrected *p* < 0.05), related to randomly assigned events, survived more often than expected by chance (i.e., more than 1 in 20). Second, Flanagan and colleagues evaluated the effect of including non-epileptic sharp EEG transients in the EEG-fMRI analysis of clear epileptic spikes (51). These events can result in physiologically plausible BOLD changes that survive a statistical threshold (in both the patient and control group). Third, An and colleagues determined sensitivity and specificity of EEG-fMRI through a different approach, using surgical outcome as “ground truth” (32). True-positive (concordance with resection zone and good surgical outcome) and false-negative (discordance with resection zone and good surgical outcome) fractions were determined in the patients, as were true-negative (discordance with resection zone and poor surgical outcome) and false-positive (concordance with resection zone and poor surgical outcome) fractions. However, poor surgical outcome could have several reasons (incorrect location of surgery, correct location but intra- or post-operative complications, partial resection of the epileptogenic zone, and no resectable epileptogenic zone), leading to equivocal interpretation of the results (52). This is the reason why we established sensitivity only in successfully operated or well-defined patients, taking the (effective or hypothetical) resection zone as central point (patients with poor surgical outcome were not included). On the other hand, specificity was defined in healthy case-controls, taking absence of epileptic activity as “ground truth.”

Different statistical thresholds (uncorrected *p* < 0.001 and FWE corrected *p* < 0.05, for instance) can result in very divergent specificities and sensitivities. This information is crucial as these thresholds are commonly reported in EEG-fMRI validation studies. We argue that EEG-fMRI outcome studies should be reported with settings that have maximal specificity. However, when the purpose of EEG-fMRI is to guide the implantation of intracranial electrodes, a high sensitivity might be preferred (17).

### The cluster containing the maximal significant activation

The presence of multiple clusters of BOLD activation raises an important question: how can we identify the cluster overlapping the IOZ in a highly specific but often widespread interictal epileptic network without prior knowledge of the IOZ? The cluster, containing the maximal significant BOLD activation, with a minimal cluster size of 350 voxels, and with a broad range of *Z*-score thresholds from 3.2 to 3.5, had 57% sensitivity and 100% specificity for localizing the IOZ, similar to the accuracy of all EEG-fMRI activation clusters. The performance of this unique cluster was robust and did not critically depend on a single *Z*-score or cluster size threshold. Our findings confirm the observations that the cluster containing the maximal significant activation is important in the localization of the IOZ (45).

We considered two other aspects of the EEG-fMRI maps. First, the maximal significant activation voxel had a lower performance for localizing the IOZ compared with the cluster containing this voxel. In some patients, this voxel was localized at the border, but just outside the IOZ, while in others, it was found more remote. Hauf and colleagues (46) ascribed similar findings of distant fMRI peak activations to the effect of propagation. Second, deactivations were only infrequently found inside the IOZ, consistent with other reports (21, 53).

### Involvement of remote regions: An epileptic network

There is a bulk of evidence that so called “focal” epilepsies are not strictly localized to well-circumscribed focal brain areas, but constitute larger epileptic networks (42, 43). When using a setting of high specificity (100%), almost half the patients with an activation overlapping the IOZ had at least one additional activation cluster in more distant areas. These remote findings can be considered as true positives. Activations at a distance have been interpreted as an extended or multifocal IOZ (10, 21). However, the presence of these remote activations did not preclude successful surgical outcome in three operated patients in our study. Therefore, spike-related BOLD clusters distant to the IOZ could also represent areas of propagated activity, as suggested by different authors (6, 25, 54, 55).

### Validity of the ictal onset zone definition

Seizure freedom and good functional outcome are the ultimate goals of epilepsy surgery. So far, 8 of the 21 patients underwent successful surgery, and we considered the resection zone as IOZ. The extent of the resection zone depends on the surgical approach and can overestimate the actual IOZ. We have shown that only about one-quarter of resected brain tissue overlapped the structural lesion or SISCOM hyperperfusion cluster (27). However, after successful surgery, sufficient brain tissue has been resected.

Thirteen patients are awaiting surgery, refused surgery, or were ineligible for surgery due to proximity of the IOZ to eloquent regions. In this non-operated group, we chose to define the IOZ as the hypothetical resection zone, based on multidisciplinary clinical consensus and regardless of eloquent cortex. As already mentioned, a prerequisite for inclusion was concordance of all modalities, including electroclinical information, structural imaging, SISCOM, FDG PET, and intracranial EEG-recordings. Multimodal concordant seizure focus localizing data increase the likelihood of benefit from surgical treatment (56–58). To avoid a rater-dependent bias in the manual delineation, we restricted the volume of the IOZ to the region of ictal hyperperfusion within this hypothetical resection zone. In our center, ictal and interictal SPECT are part of the presurgical work-up. SISCOM has several advantages: (i) it samples the whole brain, which offers an ideal comparison with the results of EEG-fMRI, (ii) it displays relative changes in cerebral blood perfusion associated with neuronal metabolic activity, (iii) a SISCOM *Z*-threshold = 1.5 results in optimal localization of the IOZ (36), (iv) it is a non-invasive test, and (v) early ictal tracer injections, as achieved in most of our patients, are known to be related to correct localization of the IOZ (59).

### Limitations

We stress that sensitivity and specificity calculations only apply to patients in whom spikes were found during EEG-fMRI. In two patients, more than one spike-type (based on topography) was found during fMRI. In these cases, we decided to determine the results driven by the most frequent spike-type during video-telemetry only, similar to Elshoff and colleagues (26). High correlations between the localization of the lobe producing the most active spiking and that of the IOZ have been found for temporal lobe epilepsies (60). Moreover, it was shown that the lobe producing the most active spiking correlated highly with the ultimately resected lobe harboring cortical dysplasia (61).

The number of successfully operated patients and their follow-up period is limited. To increase the group size, a surrogate for the effective resection zone was adopted in those patients who could not undergo surgery. This allowed us to study a representative and larger sample of patients with a well-defined IOZ after a presurgical evaluation. Studies including larger number of patients and control subjects will be required to fine-tune EEG-fMRI settings. Furthermore, this could allow subpopulations (TLE versus ETLE) to be studied, as sensitivity and specificity are presumably also dependent on brain localization.

## Conclusion

High sensitivity and specificity of spike-related EEG-fMRI for the detection of the IOZ are crucial for the clinical implementation of the technique in the presurgical planning of refractory focal epilepsy. Our data provide a guideline for different EEG-fMRI settings with their respective sensitivity and specificity for detecting the IOZ. Using optimal settings, we found that the unique cluster containing the maximal significant BOLD activation was a sensitive (57%) and specific (100%) marker of the IOZ.

## Author Contributions

Substantial contributions to the conception or design of the work; or the acquisition, analysis, or interpretation of data for the work: Simon Tousseyn, Patrick Dupont, Karolien Goffin, Stefan Sunaert, and Wim Van Paesschen; drafting the work or revising it critically for important intellectual content: Simon Tousseyn, Patrick Dupont, Karolien Goffin, Stefan Sunaert, and Wim Van Paesschen; final approval of the version to be published: Simon Tousseyn, Patrick Dupont, Karolien Goffin, Stefan Sunaert, and Wim Van Paesschen; agreement to be accountable for all aspects of the work in ensuring that questions related to the accuracy or integrity of any part of the work are appropriately investigated and resolved: Simon Tousseyn, Patrick Dupont, Karolien Goffin, Stefan Sunaert, Wim Van Paesschen.

## Conflict of Interest Statement

Dr. Stefan Sunaert has received honoraria for speaking engagements from Philips Medical Systems. Dr. Simon Tousseyn reports industry-funded (UCB and GSK) travel, not included in the study funding, to attend AES meeting, ECE, and IEC. Dr. Wim Van Paesschen received honorary for speaking engagements (GSK), serves on a scientific advisory board (UCB and GSK), reports industry-funded travel (UCB and GSK), not included in the study funding, to attend AES meeting and ECE.

## Supplementary Material

The Supplementary Material for this article can be found online at http://www.frontiersin.org/Journal/10.3389/fneur.2014.00131/abstract

## Footnotes

^1^http://fmri.uib.no/tools/bergen_plugin.htm

^2^http://www.sccn.ucsd.edu/eeglab/

^3^http://www.fil.ion.ucl.ac.uk/spm/
