# Supplementary material for: Sensitivity and Specificity of Interictal EEG-fMRI for Detecting the Ictal Onset Zone at Different Statistical Thresholds
Source: Front Neurol. 2014 Jul 17;5:131. doi: 10.3389/fneur.2014.00131 (PMC4101337; doi:10.3389/fneur.2014.00131)

***Supplementary Material***

**Sensitivity and specificity of interictal EEG-fMRI for detecting the ictal onset zone at different statistical thresholds.**

**Simon Tousseyn^1,2^*, Patrick Dupont^1,2,3^, Karolien Goffin^4^, Stefan Sunaert^2,5^, Wim Van Paesschen^1,2^**

^1^Laboratory for Epilepsy Research, UZ Leuven & KU Leuven, Leuven, Belgium

^2^Medical Imaging Research Center, UZ Leuven & KU Leuven, Leuven, Belgium

^3^Laboratory for Cognitive Neurology, UZ Leuven & KU Leuven, Leuven, Belgium

^4^Department of Nuclear Medicine, UZ Leuven & KU Leuven, Leuven, Belgium

^5^Radiology Department, UZ Leuven & KU Leuven, Leuven, Belgium

*** Correspondence:** Dr. Simon Tousseyn, Laboratory for Epilepsy Research, UZ Leuven & KU Leuven, Herestraat 49, 3000 Leuven, Belgium

simon.tousseyn@gmail.com

1. **Supplementary Data**

## Alternative approach for the calculation of sensitivity and specificity

In an additional alternative analysis (‘Method 2’), sensitivity and specificity were both determined within the patient group. Calculation of true positives, false negatives and sensitivity remained, therefore, unchanged based on the real spike onsets in patients. False positives and true negatives were now determined in the patient group based on the time onsets of the randomly permuted original spikes (1 permutation per patient). Specificity equaled the proportion of patients who lack a suprathreshold BOLD fluctuation in any part of the patient’s brain related to these ‘nonsense’ events. In Figure S1, we illustrate the ROC-curves based on the original approach (‘Method 1’) and based on the alternative Method 2. Both methods show similar sensitivity and specificity.

1. **Supplementary Figures and Tables**

## Supplementary Figures

Figure S1 illustrates the ROC-curves based on the original Method 1 using patients and controls (full lines) and the alternative Method 2 using the patients only (dotted lines): (A) EEG-fMRI activations, without constraint on cluster size; (B) EEG-fMRI cluster containing the maximal significant activation, without constraint on cluster size; (C) EEG-fMRI activations, with a minimal cluster size of 350 voxels and (D) EEG-fMRI cluster containing the maximal significant activation, with a minimal cluster size of 350 voxels.


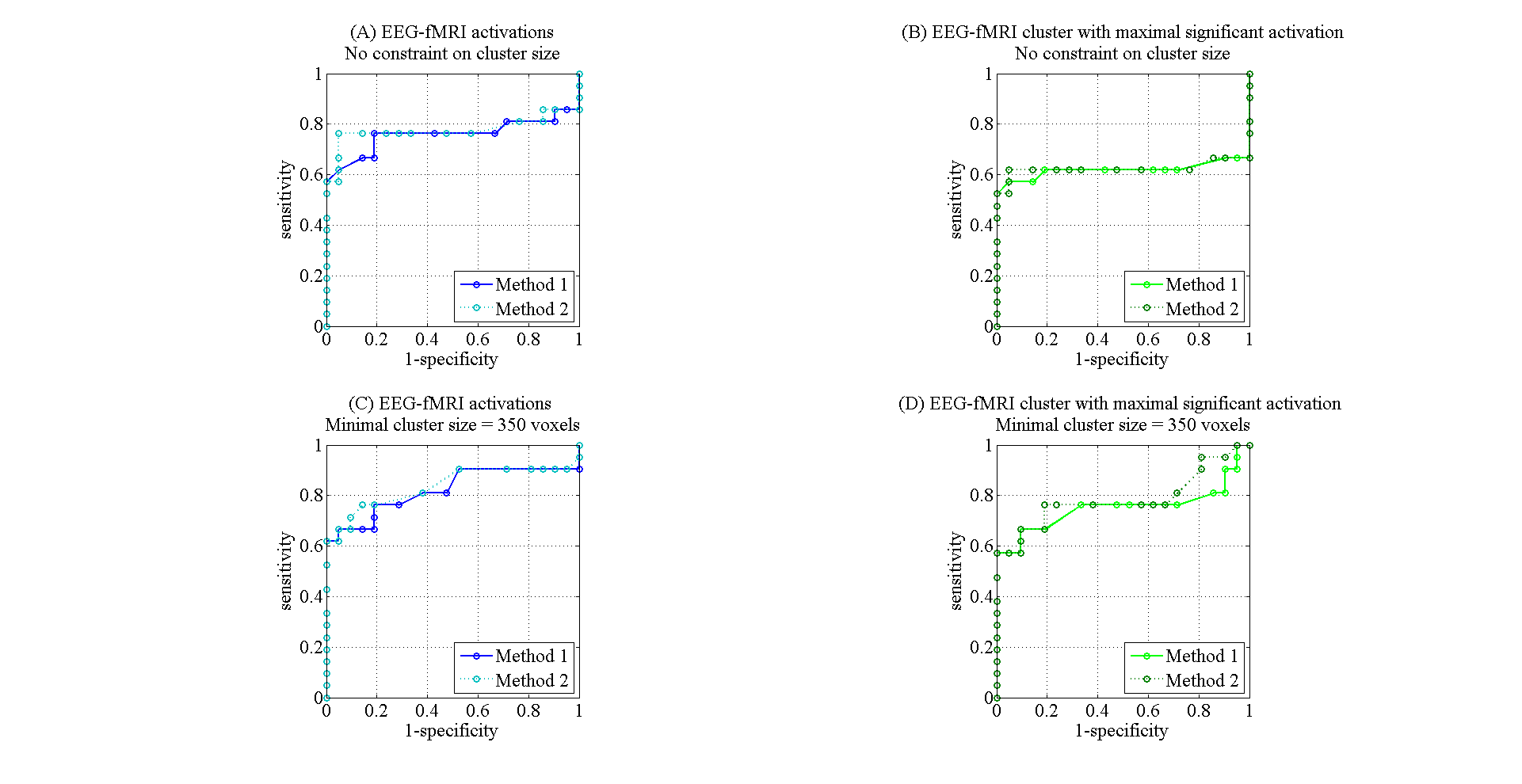

Supplement: Supplementary file 1 [file Data_Sheet_1.DOCX]
